# Relational Competence, School Adjustment and Emotional Skills: A Cross-Sectional Study in a Group of Junior and High School Students of the Sicilian Hinterland

**DOI:** 10.3390/ijerph20032182

**Published:** 2023-01-25

**Authors:** Monica Pellerone, Juan Martinez Torvisco, Stesy Giuseppa Razza, Alessandra Lo Piccolo, Maria Guarnera, Valentina Lucia La Rosa, Elena Commodari

**Affiliations:** 1 Faculty of Human and Social Sciences, Kore University of Enna, 94100 Enna, Italy; 2 Department of Psicología Cognitiva, Socialy Organizacional, Universidad de La Laguna, 38071 San Cristóbal de La Laguna, Spain; 3 Faculty of Humanities, Foreign Language and Education, Kore University of Enna, 94100 Enna, Italy; 4 Department of Educational Sciences, University of Catania, 95125 Catania, Italy

**Keywords:** social competence, school adjustment, emotional skills, family context, adolescence, COVID-19

## Abstract

Research has demonstrated the influence of emotional adjustment on the manifestation of problematic behaviors in adolescence, especially during the COVID-19 pandemic. The aim of the present research is to investigate the role of self-esteem and relational skills on school performance in a group of middle and high school students during the COVID-19 period. The research involved 392 students, aged between 11 and 20 (M = 13.78; S.D. = 2.56). Participants completed the following instruments: an anamnestic constructed ad hoc questionnaire; the Interpersonal Relationships Test, in order to evaluate the perception of adolescents concerning the quality of their relationships in social, family and school contexts; and the Multidimensional Test of Self-Esteem, structured in six scales, which coincide with the dimensions considered constitutive of self-esteem. The preliminary data have shown how the older girls, attending the high school, tend to manifest a higher level of social competence with peer group and teachers. Furthermore, the perception of a reduced emotional self-efficacy but an elevated environmental control and good interpersonal skills seem to predict the school adjustment. Understanding adolescents’ perceptions of difficulties and their social support networks can offer some insight into how major social changes can be associated with individual well-being, especially during the COVID-19 pandemic.

## 1. Introduction

Adolescence is characterized by physiological, morphological, sexual, cognitive and social changes [1]. In the literature it is defined as a “new birth” because, during childhood, while the child is interested in the external world and its phenomena, the adolescent is oriented to develop an inner and introspective representation of the world [2].

The life cycle is characterized by a series of evolutionary stages, which involve the carrying out of developmental tasks; pre-adolescence and adolescence can be placed along a continuum. Starting from pre-adolescence, in fact, individuals begin to deal with a series of tasks concerning the personal, relational and social environment in order to reorganize one’s identity [3].

With profound developmental changes, adolescents have a stronger desire for social interaction and are more sensitive to social isolation [4]; interactions with teachers, peers, and others are crucial elements in their learning experiences [5].

A primary role, in this evolutionary phase, is assumed by relational variables, which fall within the transversal skills, which appear as fundamental requirements for facing situations of great change, such as the COVID-19 pandemic, which has affected the whole world starting from March 2020. COVID-19 (acronym for coronavirus disease 2019) represents, in fact, a challenge on several fronts: health, economic, social and educational. With reference to the Italian educational context, starting from March 2020, the Government has ordered the temporary closure of schools throughout the national territory as a measure to prevent the spread of the infection [6].

The Italian schools, in compliance with the provisions of the government, have implemented remote teaching systems to allow students and teachers to complete the school year, through synchronous lessons (lessons in real time) and asynchronous lessons (the conservation and dissemination of teaching material, such as video lessons, handouts, audios and films). In particular, the government has provided funds to equip schools with digital tools and for the use of e-learning platforms, which provide varied information within a different environment, which requires students to use technology and communicate in a virtual environment effectively, resisting the distractions of the new learning environment [7,8,9]. All of this made online learning extremely challenging during the pandemic.

The new educational and socio-relational context, characterized by the distance learning process, resulting in physical, social and emotional detachment, has amplified the interpersonal and socio-emotional suffering of adolescents, compared to individuals in other stages of development. Indeed, adolescents during this pandemic phase were required to have high emotional competence to effectively deal with emotional distress, increased resilience and levels of self-esteem to face the challenges of the COVID-19 pandemic [10,11].

In accordance with the aforementioned international literature, the present study measures the role of emotional ability and relational skills, as key constructs to investigate their implications on students’ academic performance during the COVID-19 period.

## 2. Emotional Skills and Relational Competence during Adolescence

In the COVID-19 period, the family represented an important support and social network, as confirmed by a study by Rogers and Cruickshank [12] on the perception of change in the quality of relationships, which found that adolescents reported higher levels of support within the family context and slightly lower levels of conflict than before the pandemic. In general, it is possible to state that greater perceived family instability was associated with a worsening of functioning and general well-being in adolescents; an improvement was, instead, associated with high levels in overall family functioning. Data show that adolescents’ perception of change in the quality of relationships with parents is related to their psychosocial functioning during the COVID-19 pandemic. This relationship could be due to the fact that parents, through a functional parenting, contribute to the development of their children’s emotional capacities during childhood, and transmit internal operating models that will influence their way of relating to others and their self-representations, especially during adolescence.

Specifically, emotional competence is the ability to purposefully experience and express a variety of emotions, regulate expressiveness and emotional experience when necessary, and understand one’s own and others’ emotions. These skills, as they develop during childhood and adolescence, support the successful resolution of developmental tasks focused on social and academic achievement [13].

Bowlby has theorized that, during infancy, individuals internalize models of repeated interactions with their caregiver (characterized by parental control and care), which serve as a cognitive map for the management of interpersonal relationships during adolescence and adulthood [14]. The internal representations of attachment evolve over time and are likely to experience profound reorganization, consequent to both individual development and significant life experiences. Such models represent, at least partly, the base of the beliefs of self-efficacy, influencing the modality of emotional regulation, the concept of oneself and the behavioral strategies for the management of relationships [15]. In particular, research on adolescents and young adults underlines that subjects with secure attachment style manifest lower levels of negative emotions and, as a result, establish strong relationships with others—who can provide them with support during emotionally difficult situations—and a higher level of environmental control [16]. In a different way, subjects with insecure attachment style experience lower levels of positive emotions than those with secure attachments, and show inability to manage stress, anxiety, and depression, and deficits in the ability to self-regulate and in relational skills [17].

## 3. Emotional Development and Learning Processes

Learning is understood as a complex and multi-determined process, which must take into account the emotional and relational experiences of the subject in a bio-psycho-social perspective, that is, within his/her family, the peer group and the school environment. It is the quality of emotional and social relationships that influences openness and curiosity towards new experiences, and the ability to perceive connections and discover their meanings. Inadequate relationships lead to an unstable construction of reality and therefore can produce disturbances in the categories of space, time and causality; on the other hand, learning difficulties tend to cause emotional and behavioral disturbances, which increase during adolescence [18].

Emotions and learning processes are, therefore, connected concepts: learning requires thinking, and thoughts directly influence emotional experiences [19]. The link between these two dimensions is evident when evaluating self-representation, which each student possesses during the learning process. Many studies confirm the hypothesis that levels of self-esteem influence the current state of mind by the way of thinking and perceiving events, by what is remembered and by the decisions that are made [19,20]. Since we are not able to directly see the emotions experienced, these can only be deduced through behaviors, interpretable from the subjective vision of the events themselves.

The link between emotions and the learning process became even stronger during the COVID-19 pandemic, especially among teenagers. The pandemic has intensified this link because adolescents have had difficulty regulating emotions due to changes in social relationships [21,22] and have experienced higher levels of emotional distress [23].

The last three years, in fact, have been characterized by salient events for most young people, such as the closure of schools, social distancing, the use of masks and the implementation of public health guidelines; in this context, socio-emotional skills possessed by adolescents represent a protective factor for both school success and success in life. In fact, high emotional competence could not only lessen mental health issues but could also contribute to school performance in adolescent populations [24,25,26]. In detail, literature underlines that low emotional competence is related to increased mental health problems [27,28,29], which in turn interfere with academic and school performance [30,31]. Furthermore, according to recent research, students with a better ability to perceive and regulate emotions tend to manifest higher online learning readiness levels and to be more resistant to distractions [32], so they are more likely to have better school performance in an online learning setting [33,34].

Although virtual platforms have offered children and young people the possibility of some form of socially distanced connection, virtual platforms cannot replace face-to-face interaction building social and emotional skills [35,36]. Teens need social and emotional education, practice and feedback during prolonged interaction and collaboration [37].

## 4. The Role of Family Relationships, Social Competence and Self-Esteem on School Adjustment

The literature highlights how the pandemic has had a strong impact on internal family relationships [38,39]; in fact, although supportive relationships can reduce the negative effects of disasters, the quality of these relationships within the family can change due to a pandemic [40]. In particular, the extended duration of the COVID-19 pandemic and preventive measures have limited face-to-face social contacts and sources of support outside the family [41]. This absence of supportive social networks affected the social and emotional development and the quality of relationships within the family negatively, especially during adolescence [42,43]. Families have been forced to take on a new role in educating their children and have educational agents [44,45] in order to assume the roles of teachers and learning facilitators [46,47]. They have become one of the main cogs that have ensured the success or failure of education systems. On the other hand, schools have been forced to establish a dual aspect of relationship with families [48,49] and students, in order to continue developing teaching–learning processes. Likewise, families have had to internally strengthen relationships with their children in order to optimize educational processes.

Consequently, adolescents’ motivation to study was strongly influenced by both the fact that parents acquired the role of teachers and by the need to learn all the technological skills necessary to be able to access distance learning quickly. One of the most considerable challenges traditionally tackled by teachers is the commitment to forging stronger bonds between the school and the families and between the students and family [50], by opting for more significant family involvement, which becomes, under certain circumstances, a source of social support for adolescents.

Within this new scenario, for many parents it has been a source of stress to reconcile the different roles to be covered as a full-time (parent, spouse, worker and teacher) with a consequent increase in anxiety symptoms, sleep disturbances, negative thoughts and irritability. For some parents it may have been particularly tiring to maintain private spaces and autonomy for themselves and for their children: some may have reacted to the reduction of the physical spaces of autonomy by intensifying control behaviors towards their children, monitoring more assiduously their school conduct and use of social networks; others may have implicitly asked their children for more adult, responsible and independent behavior to manage their daily lives; still others may have exercised their parental authority more punitively or harshly.

The effects of the pandemic have also been multiple for children: similarly to parents, children too may have reacted to isolation and forced withdrawal from social life by showing an increase in behavior that is either more infantile and regressive, with greater dependence on the parental figures, or on the contrary more aggressive, conflicting and distancing, with consequent massive use of social media; in the latter case, research has found, in adolescents in particular, an increase in the sensation of lack of air and a significant alteration of the sleep–wake rhythm, together with an increased emotional instability, irritability and changes in mood, as a result of excessive Internet use [51].

The increase in the use of social media—to follow school lessons and maintain contact with family and friends—has resulted in an increase in social media disorder symptoms compared to peers before the pandemic, above all among older adolescents. Adolescents’ problematic use of social media has also been linked to their higher emotional-behavioral symptoms and disordered eating behaviors; specifically, older adolescents reporting more social media disorder symptoms were more likely to experience learning difficulties, perhaps due to excessively focused attention on social media, which may affect the adolescent’s ability to pay attention to other aspects of life, such as face-to-face relationships with family members and schoolwork [52].

In this regard, the meta-analysis by Vannucci [53] appears interesting, in which an association between the use of social media and greater consumption of drugs, general risky behaviors and dysfunctional relationship with adults and peers during late adolescence is reported, compared to younger teenagers. The lack of positive feedback obtained online, in fact, can have harmful effects on well-being as they can elicit a feeling of rejection, the belief of being excluded or ignored, giving rise to a higher negative affectivity dominated by feelings of sadness, anxiety, stress, anger or embarrassment.

Furthermore, recent research shows that social support plays a key role not only in emotional exhaustion but also in adolescent academic achievement, a role often mediated by self-esteem [54]. In fact, social relationships can enhance students’ experience of being appreciated and accepted, increasing the level of self-esteem and the perception of well-being [21]. Thus, self-esteem, which refers to an individual’s overall emotional assessment of their own worth, is related to the individual’s emotional experience; positive emotions tend to promote the development of self-esteem, while negative emotions can weaken the level of self-esteem.

Self-efficacy and self-esteem are consequential psychological concepts with respect to student life, and both are strongly associated with student achievement and their self-regulation and motivation [21,55,56]. Although the psychological constructs of self-efficacy and self-esteem both concern cognition and self-perception, they are distinct in the sense that self-efficacy describes one’s optimistic perception of one’s competence beliefs and confidence [57], whereas self-esteem reflects the affective evaluation of one’s self-worth [58]. Self-efficacy can be regarded as a positive resistance and resource factor when coping with adversity [59]; instead self-esteem has been associated with coping, reflecting how people with low levels of self-esteem relied on emotion-based coping strategies, while people with high levels of self-esteem relied on beneficial coping strategies directed at problem solving. Therefore, self-efficacy and self-esteem may help students cope with psychological challenges; one’s perception of abilities and limitations represent an important internal resiliency factor during a crisis phase and these aspects of self-perception are shaped by social support. That is, a decrease in social support might negatively affect a student’s self-perception as a coping resource, and that could in turn deteriorate their well-being, above all during the pandemic [60].

Research indicates that both self-efficacy and self-esteem are shaped by social relations. In detail, watching others perform tasks and comparing others’ and one’s own abilities, as well as receiving encouragement from other people, reflect how social support can shape the individual’s sense of self-efficacy. Adolescents strive to maintain interpersonal relationships; consequently, self-esteem fluctuates as a function of the person’s perception of being valued and accepted by others, but also in relation to age. In fact, the literature [61,62] confirms the presence of a reduction in self-esteem levels during adolescence, attributing this decline to maturational changes associated with puberty, to cognitive changes associated with the emergence of formal operational thinking and to socio-contextual changes associated with the transition from one school order to another. Erikson’s model of psychosocial development suggests that, as individuals age, they become more accepting of who they are; in particular, while in adulthood they develop the “integrity of the ego” and a general acceptance of the realizations of their life. This acceptance of the self does not occur in adolescence, a phase characterized by an initial structuring of identity. Furthermore, in late adolescence, teens invest in new domains at the expense of other domains, and become more accurate in measuring their relative strengths and weaknesses in distinct areas of functioning [63]; this could lead to a temporary reduction in self-esteem levels in adolescence both in the internal components of self-esteem (emotional competence and body image perception) and in the external ones (interpersonal relationships, school success and environmental control).

For these reasons it seems essential to examine all the privileged contexts which allow the adolescent to structure his/her own identity, such as the family environment, the peer group and the school context. In particular, studies have indicated that school affiliation may influence not only academic achievement but also self-concept and self-efficacy, school-related emotions and control over the school environment, resulting in satisfaction and greater adjustment [64].

In accordance with the Self-Determination Theory, it is important to underline that to maximize learning motivation in adolescents it is necessary to satisfy students’ needs for relationships and connections in the school context [65]. Lazarus, in particular, has suggested that emotional capacities make individuals aware of important features of their environments and of direct cognitive processes that result in greater control of their surroundings. Furthermore, according to the cognitive-motivational relational theory [66], the emotions that direct actions and behaviors derive from their relational meanings. An individual’s interpretation of the relational significance of a specific encounter is based on an assessment of the personal consequences (positive and negative) of the relationship between environment and self. Therefore, it is possible that school belonging—which can be seen as a student’s evaluation of the positive and negative aspects of his school belonging—can influence learning-related behavior through positive learning-related emotions and the resulting perception of control on the school environment.

In line with the socio-cognitive perspective on human development, Pekrun [67] proposed that students’ cognitive assessments of academic emotion are shaped by social and environmental factors. Empirical research on the social and environmental antecedents of academic emotion has indicated that classroom factors, including the quality of education and social affiliations in the classroom and/or teacher and peer support, may influence students’ school emotions. Furthermore, the theoretical framework of Pekrun suggested that students’ positive evaluations of their social environment induce positive school emotions, which in turn influence learning and achievement; in other words, Pekrun and colleagues [68] proposed that positive emotions and the consequent perception of greater environmental control may mediate the impact of social factors on student learning, especially in adolescence.

## 5. Research Goals and Hypothesis

The present research investigates the relational competence; the level of self-esteem manifested in the family, relational, school context; and the emotional skills of a group of middle and high school students from the Sicilian hinterland.

The survey allowed verifying the importance of the concept students have of themselves during adolescence, a period of crisis represented by transitions and choices typical of this phase; in this circumstance, their self-dimension, which also includes concepts of individuation, identification, esteem, and acceptance, represents a fundamental element for its influence on the subject’s psychological well-being and way of living and representing social relationships.

According to the literature, we hypothesized that:

**H1:**  *The perception of relational difficulties could be predicted by emotional distress*;

**H2:**  *The adaptive relationship with parents could be predictive of the perception of greater environmental control in all group of participants*;

**H3:**  *The emotional ability and the perception of environmental control could be predictive variables to the perception of school success*;

**H4:**  *Emotivity and environmental control could mediate the relationship between relational competence and academic success*.

## 6. Research Method

### 6.1. Participants and Procedures

The present research involved a group of students attending Sicilian middle and high schools: 392 students, among which 158 were boys (40.3%) and 234 girls (59.7%), aged between 11 and 20 (M = 14.78; S.D. = 2.56), of which 7.7% have repeated the school year once. The students involved in the research belong to schools in the province of Gela, a Sicilian territory with a high percentage of hardship and early school leaving. In particular, the observatory on non-compliance and educational emergency of the Sicily Region has recently highlighted that in the 2021/2022 school year, from primary to middle and high schools, the data relating to evasion, abandonment and school refusal of Gela students are higher than in the whole Sicilian territory. In fact, if the regional average incidence of early school leaving for primary schools stands at 0.44%, in Gela this percentage rises to 1.2%. Similarly, in the middle school, the regional average is 5.49% against 7% for Gela. Finally, for the high school the percentages vary from 14.6% to 19% of the figure for Gela. The relevance of the phenomenon and the complexity of its implications therefore urgently require a precise definition and analysis of the problem.

### 6.2. Instruments

The instruments handed out to the group of students were the following: an anamnestic questionnaire constructed ad hoc, the Interpersonal Relationships Test (TRI) and Multidimensional Test of Self-Esteem (TMA).

The Interpersonal Relationships Test (TRI) developed by Bracken [69] is aimed at evaluating the perception of children and adolescents concerning the quality of their relationships in social, familial and school contexts. The questionnaire is divided into three sections concerning relationships with parents, peers and teachers. The Cronbach’s alpha coefficient, a measure of reliability or internal consistency, is equal to 0.97; in the present study, the Cronbach’s alpha coefficient is equal to 0.87.

The Multidimensional Test of Self-Esteem (TMA), created by Bracken [70], is based on the assumption that self-esteem is a behavioral and cognitive pattern that develops according to the learning principles. The tool, made up of 150 items evaluated on a 4-point Likert scale (absolutely true; true; not true; absolutely not true), is made up of dichotomous statements (positive and negative). The test is structured in six scales, which coincide with the dimensions considered constitutive of self-esteem, plus a total scale, namely: Interpersonal Relationships, Emotional Competence, Control over the Environment, School Success, Family Life and Body Image Perception. For research purposes we have evaluated the following scales: Emotional Competence, Control over the Environment and School Success. The Cronbach’s alpha coefficient is equal to 0.92; in the present study, the Cronbach’s alpha coefficient is equal to 0.83 for Emotional Competence Scale, equal to 0.82 for Environmental Control, and equal to 0.78 for School Success.

School authorities’, parents’ and involved students’ consensus was requested before handing out and collecting tools. Questionnaires were anonymous. The tools were administered online with the help of Google modules.

## 7. Data Analysis

In reference to the preliminary analysis, a Pearson’s correlation was carried out in order to analyze the relation between all dependent variables; the Independent Samples *t* Test analysis was conducted in order to compare emotional ability and self-esteem in both groups of students (middle and high school). In order to measure the influence of independent variables on the dependent variables, the MANOVA (Multivariate Analysis of Variance) is carried out.

A separate hierarchical regression analysis was carried out in order to evaluate the predictive role of emotional difficulties in the relational discomfort and the predictive function of adaptive relationships with parents on the perception of environmental control in all groups of participants.

Another separate hierarchical regression analysis was carried out in order to evaluate the predictive variables of the school adjustment in adolescents, including in the model of the relational ability and the perception of environmental control.

Finally, an analysis of multiple mediation with two mediating variables forming a causal chain was carried out through PROCESS v. 4.2 (Model 6): in detail, we assessed the relationship between relational competence (independent variable) and academic success (dependent variable) through the serial mediation with emotional skills and environmental control variables.

## 8. Results

In reference to the school grade, participants are divided in two groups:

- a group of 217 students attending the middle school, of which 83 were males (38.2%) and 134 females (61.8%), with ages between 11 and 14 years (M = 12.52; SD = 0.98);
- a group of 145 students attending the high school, of which 55 were males (37.9%) and 90 females (62.1%), with ages between 14 and 20 years (M = 16.4, SD = 2.02).

With reference to the first group (middle school students), the Pearson correlation analysis shows the presence of a relation between all dimensions of the TRI (Table 1).

Another correlation analysis underlines the same results in the second group (Table 2).

In reference to the level of self-esteem, the Independent Samples *t* Test analysis was conducted in order to compare emotional skills in both groups of students; items from which a significant difference emerged are included in the Table 3.

The same analysis was conducted in order to compare the perception of school success in both student groups; Table 4 shows items from which a significant difference emerged.

The Independent Samples *t* Test analysis was conducted in order to compare the peer relational skills in both groups; significant items are included in Table 5.

The same analysis was conducted in order to compare the perception of the relationship with the mother in both groups; significant items are the following (Table 6):

In reference to the relationship with the father in both student groups, Table 7 shows only significant items.

The same analysis was conducted in order to compare the perception of the relationship with teachers in the student group; significant items are included in Table 8.

In order to measure the influence of independent variables on the relational ability the MANOVA was carried out; the analysis underlines that age influences all relational variables, but gender influences only peer relationships; furthermore, the school grade seems to influence the relationship with mother, and the interaction between age and gender influences all relational abilities (Table 9). In particular, the analysis of the average scores shows how the older girls, attending the high school, tend to manifest a higher level of social competence with peer groups (M = 3.38, S.D. = 0.47) and with teachers (M = 3.2; S.D. = 0.53); 16-year-old females (attending the high school) show a better relational competence with their mothers (M = 3.94; S.D. = 0.25); 19-age males (attending the high school) manifest a good relational competence with their fathers (M = 3.17, S.D. = 0.37).

In order to measure the influence of independent variables on the environmental control, MANOVA was carried out; the analysis underlines that the interaction between age and gender (F = 1.97, *p* < 0.05) influences environmental control. In particular, the post hoc analysis shows how 18-year-old males seem to have a better perception of environmental control. It is interesting that independent variables do not seem to influence the school success (*p* > 0.05).

In reference to the first hypothesis, the first separate hierarchical regression analysis shows that the emotional difficulty is a predictive variable of the relational discomfort with peer group (β = 0.25, *p* > 0.001), explaining 20% of the overall variance; similarly, it is predictive of the relational discomfort with teachers (β = 0.21, *p* > 0.01), explaining 25% of the overall variance. Data seem to confirm the research hypotheses.

In reference to the second hypothesis, hierarchical regression shows that only an adaptive relationship with the mother is predictive of the perception of better environmental control (β = 0.26, *p* > 0.05), explaining 32% of the overall variance. Data seem to partially confirm the second hypotheses.

Another regression analysis was carried out in order to analyze predictive variables of school success in all groups of students, including in the model the perception of emotional ability and environmental control, and the general level of relational skills. Data show that a reduced emotional ability but an elevated environmental control and a high level of relational skills seem to predict the school success in all students (Table 10).

The same data analysis was carried out in order to analyze the predictive variables of school success including all components of relational skills: that is, relationships with peer group, teachers and parents. The data underline the predictive role of emotional ability, environmental control and relationship capability with the peer group and the father; in particular having the perception of a reduced emotional ability but an elevated environmental control, and having positive relations with the peer group and father is predictive of high school success (Table 11). The data seem to partially confirm the research hypothesis.

Finally, the study assessed emotivity and environmental serial mediation of the relationship between relational competence (independent variable, IV) and academic success (dependent variable, DV). An analysis of multiple mediation with two mediating variables forming a causal chain was carried out through PROCESS v. 4.2 (Model 6) was used to test the hypothesis model. Once the linear regression analysis was performed using the PROCESS macro inserted in the SPSS statistical package, the results revealed that, firstly, the IV (independent variable), relational competence, has a significant impact on MV1 (mediating variable) emotivity (β = −0.3801, t = −5.636, *p* < 0.001), and IV has a significant impact on MV2, environment (β = 0.2582, t = 2.789, *p* < 0.005); emotivity has a significant impact on environment (β = −0.2280, t = −3.411, *p* < 0.001). Furthermore, relational competence has a significant impact on DV (dependent variable) academic success (β = 0.2179. T = 5.371, *p* < 0.001); emotivity was found to have significant impact on academic success (β = −0.3259, t = −11.073, *p* < 0.001). Environment was also found to have a significant impact on academic success (β = 0.0888, t = 4.036, *p* < 0.001). The total effect of relational competence on academic success through mediating variables, emotivity and environment was found to be significant (β = 0.3723, t = 8.143, *p* < 0.001). Regarding indirect effects (bootstrapping), there are three with 95%: the first one represents the first mediating variable (emotivity) between relational competence and academic success (β = 0.1239, SE = 0.0297, t = 4.171, CI [0.0710; 0.1884]), the second indirect effect of environment mediates between relational competence and academic success (β = 0.0229, SE = 0.0111, t = 2.063, CI [0.0047; 0.0480]), and the third indirect effect shows the relation between relational competence and academic success through the mediating variables of emotivity and environment as serial variables (β = 0.0077, SE = 0.0038, t = 2.026, CI [0.0019; 0.0164]).

**Figure 1:**
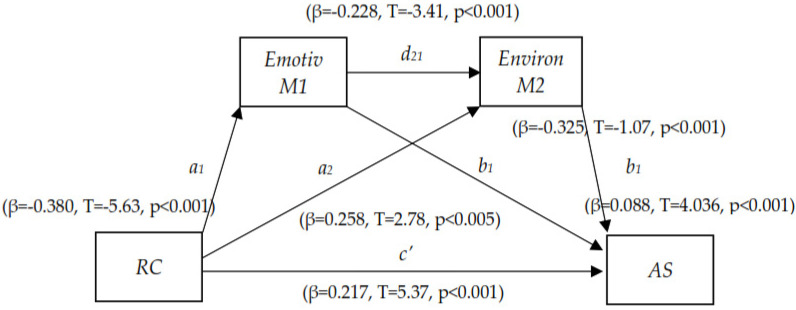
Coefficients of the relationship between mediating, criteria and predicted variables. Note: Indirect effect of *X* on *Y* through *M_i_* only = *a_i_ b_i_*; Indirect effect of *X* on *Y* through *M_i_* and *M_2_* in serial = *a_i_ d_21_ b_2_*; Direct effect of *X* on *Y* = *c’*. Abbreviation: RC, Relational Competence; AS, Academic Success.

Furthermore, to determine which of these effects is more significant, a pairwise contrast of indirect effect was performed. The third contrast was found to be non-significant (β = 0.0152, SE = 0.0111, t = 1.369, CI [−0.0052; 0.0393]). Hence, relational competence has a higher effect on academic success through emotivity than it does through its effects on emotivity and environment serially joined and, subsequently, there is a partial serial mediation of emotivity and environment on the relationship between relational competence and academic success. Mediation summary is presented in Table 12, and Figure 1 shows the coefficient values from the relationship between mediating, criteria and predicted variables.

Regarding the model summary total effect, it reveals a value of R-Sq of 0.1453; that is to say, a 14.53% of the variance is explained by the model (MSE = 0.0721. F = 66.309. df = 390. *p* < 0.000).

## 9. Discussion

There are numerous and frequent problems and shortcomings that adolescents show every day both in the family and at school as well as in their free time: anxiety, depression and isolation, poor attention and concentration skills, difficulty in managing emotions, inadequacy in management of interpersonal relationships, lack of sense of responsibility, and tendency to impulsiveness.

The most emotional competence studies in adolescence have been conducted in traditional face-to-face learning contexts and have focused on specific emotions. For this reason, the present research investigated the role of emotional and socio-relational competence in online learning contexts, during the current pandemic. In particular, although the emotional competence plays different roles in the lives of adolescents and young adults [71], few studies have differentiated the roles emotional competence plays in school adjustment and achievement among adolescents (middle and high school students).

The present research has investigated the relational competence, the level of self-esteem in the family, relational and school context and in emotional skills in a group of middle and high school students from the Sicilian hinterland. In reference to the level of self-esteem, the Independent Samples *t* Test analysis has shown how older children (attending high school) tended to have a reduced self-evaluation, both in the perception of their own emotional skills, academic success and relational skills. This result confirms the literature that underlines how, in late adolescence, individuals are strongly influenced by social relationships, which affect the representation of the self and, therefore, the level of self-esteem; moreover, the presence of a reduction in self-esteem levels during the late adolescence could be a consequence of the maturational and cognitive changes associated with puberty, but above all of socio-relational and contextual changes associated with the transition from one school level to another.

The Multivariate Analysis of Variance has shown how older girls, attending high school, tended to manifest a higher level of social competence with their peer group and with teachers, but not with their parents. The difference linked to the age and gender variables may be associated with the type of relationship that adolescents have with their parents; it is possible that parents are more accepting and lenient towards young adolescents; on the other hand, parents could be more rigid and less accepting of older adolescents; this is consistent with the increase in relational asymmetry between parent and child through late adolescence.

In different way, 16-year-old females showed a better relational competence with their mother and 19-year-old males a good relational competence with their father. These data confirm the literature which underlines how, in late adolescence, it is the relationship with the father (not the one with the mother) that has an effect on self-esteem; it is above all the father’s encouragement towards autonomy and independence that helps the child to increase their self-esteem with respect to interpersonal relationships, family life and total self-esteem.

Confirming the first hypothesis, emotional difficulty is a predictive variable of the relational discomfort with the peer group; similarly, confirming the second hypothesis, the presence of an adaptive relationship with the mother is predictive of the perception of better environmental control. Data validate the research on adolescents and young adults, underlining that subjects with secure attachment style manifest lower levels of negative emotions and, as a result, establish strong relationships with others and a higher level of environmental control. Therefore, starting from a bond of functional attachment with the parental figures, the adolescent implements an identity reflection, which allows them to monitor the emotional processes and the relational dynamics from which their thoughts and actions move, accentuating the feeling of perceiving themselves as “captain of their own life”.

Furthermore, confirming the third research hypothesis, the data showed that the perception of a reduced emotional self-efficacy but elevated environmental control and good interpersonal skills seem to predict school success. This result seems to confirm that part of the literature which underlines how a greater ability to control the external environment has a positive influence on school performance: for example, think of the effect of a compliment on a person who believes he can control events; it can increase the motivation linked to the pleasure of doing that activity or, on the contrary, the same compliment towards a person who feels at the mercy of events further can reduce the motivation linked to pleasure [72]. These data reflect the individualistic culture of belonging, which can have an influence on the development and structuring of self-esteem; in particular, within the individualistic cultures, such as today’s Western ones, the focus is greater on the tendency to believe in one’s ability to influence the situations and events of one’s life, somehow feeling in power to change the environment.

Regarding the predictive value of low emotional self-esteem on academic success, this could be an indicator of the presence of thinking oriented towards the outside and towards the task. Outward-oriented thinking, in particular, reflects a marked attention to the details of external events, people and places rather than to internal experiences, including how the person reflects on his or her feelings and uses them as guides for one’s thoughts and own behaviors; this could lead to a low perception of one’s emotional capacities in adolescence. With regard to the relationship between “task orientation” and scholastic success, the literature underlines how it is characteristics such as perseverance, low distractibility and a good level of activity that determine high scholastic success; on the contrary, a reduced level of activity or low perseverance in completing a task are elements that compromise the ability to pay attention and concentrate and, therefore, negatively influence performance and the achievement of a goal. Even the characteristics belonging to a temperamental scheme called “personal social flexibility”, such as quality of mood, approach/avoidance towards new situations and adaptability, were found to be correlated with school success, albeit to a lesser extent. The latter, rather than correlating with the actual performance of the pupils, in fact, contribute to the formation of teachers’ judgments on the pupils’ abilities and expectations regarding their school performance, with the risk of becoming self-fulfilling prophecies.

Finally, the mediation analysis showed the total effect of relational competence on school success through the mediation of the variables of emotional self-esteem and environmental control; with reference to the indirect effects, it appears interesting that the first is represented by emotional self-esteem in the relationship between relational competence and scholastic success, the second indirect effect is represented by the environment and the third indirect effect shows the relationship between relational competence and scholastic success through variables using emotion and environment as serial variables. By comparing these independent effects, the data showed that relational competence has a greater effect on academic success through emotion rather than through its effects on emotion and environment serially combined.

Data confirm a study conducted on 272 students, which investigated the relationships and influences between on communication and social competence, emotional intelligence and self-efficacy; the data showed a significant positive effect of social competence on academic self-efficacy, a significant positive effect of emotional intelligence on self-efficacy and, above all, a moderating effect of emotional intelligence on the relationship between communication competence and self-efficacy [73]. Similarly, a recent study conducted by the CNR (Italian National Research Council) aimed at a sample of 201 subjects (113 males and 88 females) aged between 11 and 18, which investigated the level of self-esteem, the type of relationship experienced by the adolescent within the family with the father and mother and the motivation to study. Data analyses have shown that the factor that, more than others, can predict academic performance is the level of self-esteem that the student has with respect to their academic and relational skills, a level that strongly influences grades in all subjects. Conversely, self-esteem in family relationships affects children’s self-esteem rather than having a direct impact on school performance.

Therefore, the reference points which the child can leverage to draw security take on considerable importance and which can be represented both by internal factors (feeling competent in a particular activity or interpersonal skill) or by external factors (family, teachers and companions). According to Susan Harter [74], in fact, both “instrumental” success and social approval are the cornerstones of the emotional processes associated with self-esteem. Self-assessment is strongly affected by the judgment expressed by others, so much so that it is referred to as “the image of oneself in the mirror”: it is therefore essential that the parent or educator is able to convey to the child a positive idea of their ability and demonstrate a deep confidence in their potential.

## 10. Conclusions

It is clearly known that adolescence is an extremely complex period for different reasons and in relation to different dimensions of the existence of boys and girls; what we intend to emphasize is the strong impact that this transition period has on emotional well-being and how social changes, as a whole, aggravate or facilitate some processes of psycho-physical maturation. Interpersonal relationships are one of the elements that most influence their emotional well-being and, therefore, good social relations with their peers are very important. At the same time, the presence of adult figures who show attention to the needs of children and who show concern for them and believe in them is also fundamental. It is therefore interesting to note that all those who play a role in the life of a teenager, from family members to teachers, from sports coaches to neighbors, can have an influence on those “symptoms” of emotional well-being. Therefore, there is a close relationship between the emotional well-being reported by adolescents and the family structure and the presence at school of an adult considered as important.

Experiences of neglect and antipathy in the relationship with “caregivers” and a dysfunctional educational style in family relationships predispose young people to the development of low self-esteem and psycho-social distress as a consequence of bullying and youth cyberbullying.

To solve the problem of dysfunctional parenting, it is necessary to make an educational change in the family and in the school, through a flexible program and collaboration between parents and teachers. The empowered teacher should emerge and materialize, and be able to intervene in all cases that generate a low level of self-esteem.

In light of what has been said so far and what has been confirmed by the literature on the subject, we can further affirm how the dimension of self-esteem is one of the dimensions most directly related to the physical, psychic and social well-being of the adolescent and how important it is to invest in the promotion of effective paths in this direction, at school as in the family.

More generally, it can be said that there is a prevailing need to promote educational paths that aim at the enhancement of social and prosocial skills which place the healthy emotional development of the person at the center of their interest, from first experiences and in formal contexts, and to invest in the training of effective, trained and aware educational reference figures.

The educational relationship becomes a factor of protection when it is able to provide support and when it promotes some elements in education that are already in themselves protective factors such as skills for life, self-esteem, self-efficacy, optimism and the search for meaning. An element of good family functioning is represented by the quality of relationships, which allows children to develop trust and favors the skills necessary to face this transition period. Other fundamental aspects are the support and control exercised by parents, who must first and foremost support their children on an emotional level and in activities they carry out outside the family; they must also create open communication and tolerate possible disagreements [17].

It is also appropriate that parents also provide precise guidance and clear points of reference to encourage the assumption of responsibility and limit risky behavior. A good level of parental support allows the adolescent to turn confidently outwards and makes them able to explore new roles and aspects of themselves because they know they can count on the stable emotional presence of the parents. In adolescence, parental support also includes the willingness of parents to listen to their child, to share problems and to communicate openly with them. In fact, the possibility of open discussion with parents allows adolescents to perceive themselves as loved and accepted and to develop more positive self-image. All good relationships foster the development of feelings of well-being and self-satisfaction. These relationships facilitate the academic success of the adolescent and this favors the construction of a positive identity and facilitates the overcoming of feelings of anxiety and worries related to growth, which are often part of the discomfort that the young person is facing. Many cognitive, emotional, motivational and relational factors affect adolescent academic success. A notable role is played by self-efficacy in school success and in regulating learning.

Teachers aware of the importance of their educational role can enhance their students’ cognitive skills and personal and socio-relational skills related to these activities, working to increase students’ self-efficacy and their ability to use problem-solving strategies, especially in an area where the levels of hardship and early school leaving are very high. Early school leaving in Sicily can in fact take various forms: failure to complete compulsory schooling, dropouts and irregular attendance, up to involving the subjective dimension of disappointment due to the mismatch between personal aspirations and results achieved.

One of the characteristics that the educator must promote in adolescents is self-esteem, which plays a significant role in the construction of personal identity. Adolescents who have low self-esteem fail in this and consider themselves inferior to their peers. The risk is that an adolescent, after failing to obtain significant results in socially positive environments, to defend his self-esteem seeks possibilities of success in deviant and destructive behaviors appreciated within his own group.

Moreover, experiencing a sense of inadequacy connected to school failure, together with being the object of negative evaluations and the inability to respond to the expectations of teachers and parents, can lead the adolescent to abandon school or to seek alternative sources of self-enhancement. It is clear, therefore, that the importance of creating good educational relationships can become a factor of protection for adolescents [75].

If adolescence is the most critical period of life, being a teenager during the pandemic is a huge risk factor. All the potential that young age brings with it can become a risk factor to develop many problems. Not surprisingly, all the literature taken into consideration so far shows an increase in psychological disorders in the young population. Adolescents during the first lockdown, and then throughout the course of the pandemic, developed symptoms of psychophysical suffering, which was sometimes serious, such as depression, anxiety and strong stress, which had never occurred before on such a large scale. The malaise experienced by adolescents has become of great concern to experts and this has prompted them to conduct numerous research studies to better understand their needs and discomforts. The psycho-physical health situation of adolescents is aggravated by other collateral causes, such as the socio-economic situation, the housing condition, the quality of intra-family relationships, having had first-hand experience with the virus and much more.

From what can be seen from after reading the literature, the most effective solution is to build and design a personalized intervention plan adapted to the individual in order to offer them as many tools and opportunities as possible to improve themselves and improve the relationship with others, another aspect strongly compromised during the pandemic in order to glimpse a future perspective. However, we must not mistakenly think that this is enough; it is necessary that society, institutions, educators and especially the family are all promoters of a common goal, which places as a focal point the young and as a corollary all their intrinsic and extrinsic aspects, in order to make them autonomous and the leader of their lives, which are still in the making.

## Figures and Tables

**Table 1:** Correlation analysis between dimension of TRI in the middle school students.

Variables	A	B	C	D
A. Peer relationship	-			
B. Relationship with teacher	0.564 **	-		
C. Relationship with mother	0.229 *	0.362 **	-	
D. Relationship with father	0.197 *	0.336 **	0.658 **	-

Note: ** = *p* < 0.01; * = *p* < 0.05.

**Table 2:** Correlation analysis between dimension of TRI in the high school students.

Variables	A	B	C	D
A. Peer relationship	-			
B. Relationship with teacher	0.783 **	-		
C. Relationship with mother	0.647 **	0.592 **	-	
D. Relationship with father	0.580 **	0.532 **	0.742 **	-

Note: ** = *p* < 0.01.

**Table 3:** The Independent Samples *t* Test: significant items of Emotional Competence Scale.

Items	Middle School Students	High School Students	*t* Test for Independent Group	
	M (SD)	M (SD)	T	*p*-Value
I like my life	3.38 (0.59)	3.15 (0.81)	3.09	0.00
I am not a happy person	1.77 (0.74)	2.16 (0.78)	−4.65	0.00
I feel like a failure	1.79 (0.69)	1.96 (0.84)	−2.00	0.05
I have good self-control	3.06 (0.77)	2.88 (0.79)	2.05	0.04
I often disappoint myself	2.46 (0.80)	2.65 (0.78)	−2.14	0.03
My life is unstable	1.89 (0.65)	2.14 (0.80)	−3.00	0.00
Sometimes it seems to me that I am worth nothing	2.31 (0.89)	2.62 (0.92)	−2.88	0.00
I am not as happy as I look	2.18 (0.79)	2.58 (0.89)	−3.86	0.00

**Table 4:** The Independent Samples *t* Test: significant items of School Success Scale.

Items	Middle School Students	High School Students	*t* Test for Independent Group	
	M (SD)	M (SD)	T	*p*-Value
I am good at math	2.91 (0.86)	2.64 (0.89)	2.48	0.014
Studying is difficult for me	1.94 (0.61)	2.20 (0.72)	−3.14	0.002
I am proud of my school work	3.26 (0.61)	2.99 (0.75)	3.15	0.002
I learn easily	3.15 (0.56)	2.94 (0.72)	2.48	0.01
The sciences are easy for me	2.91 (0.64)	2.70 (0.76)	2.21	0.03
My teachers have a low opinion of me	1.84 (0.61)	2.10 (0.80)	−2.72	0.01
Most of the subjects are pretty easy for me	3.19 (0.54)	2.94 (0.69)	2.02	0.00

**Table 5:** The Independent Samples *t* Test: significant items of Peer Relationship Scale.

Items	Middle School Students	High School Students	*t* Test for Independent Group	
	M (SD)	M (SD)	T	*p*-Value
I am truly understood by …	3.18 (0.56)	2.87 (0.74)	3.19	0.002
I like spending time with …	3.25 (0.53)	3.05 (0.68)	2.11	0.036
I am treated with justice by …	3.03 (0.57)	2.70 (0.79)	3.24	0.001
I am treated with justice by …	3.03 (0.75)	2.76 (0.79)	2.19	0.030
I am treated with justice by …	3.10 (0.67)	2.85 (0.81)	2.19	0.029
I am treated with justice by …	2.69 (0.78)	2.33 (0.79)	2.92	0.004
I am treated with justice by …	3.20 (0.59)	2.98 (0.65)	2.30	0.023

**Table 6:** The Independent Samples *t* Test: significant items of Mother Relationship Scale.

Items	Middle School Students	High School Students	*t* Test for Independent Group	
	M (SD)	M (SD)	T	*p*-Value
I am truly understood by …	3.40 (0.64)	3.22 (0.83)	2.29	0.02
I like spending time with …	3.40 (0.64)	3.22 (0.83)	2.14	0.03
If I was bothered by the behavior of a friend, I would tell …	3.19 (0.69)	3.00 (0.92)	2.13	0.03
I am treated with justice by …	3.25 (0.74)	2.94 (0.87)	3.63	0.00
If I were worried about a friend starting to take drugs I would say to …	3.15 (0.72)	2.70 (0.96)	5.01	0.00
When I am alone I look for the company of …	3.09 (0.74)	2.82 (0.94)	2.99	0.00
I can express my true feelings when I am with …	3.14 (0.74)	2.94 (0.89)	2.37	0.02
My happiness is greatly influenced by …	2.93 (0.78)	2.83 (0.93)	1.11	0.00
I find it hard to be myself when I’m around …	2.09 (0.88)	2.18 (0.81)	−0.96	0.01
My values are similar to …	2.88 (0.76)	2.82 (0.87)	0.66	0.02
I am motivated to give the best of myself by …	3.34 (0.67)	3.16 (0.83)	2.29	0.01
When I have worries about the future I talk about it to …	3.26 (0.67)	3.04 (0.92)	2.63	0.01

**Table 7:** The Independent Samples *t* Test: significant items of Father Relationship Scale.

Items	Middle School Students	High School Students	*t* Test for Independent Group	
	M (SD)	M (SD)	T	*p*-Value
I am truly understood by …	3.29 (0.69)	3.10 (0.85)	2.35	0.02
I am treated with justice by …	3.20 (0.71)	2.95 (0.88)	2.86	0.00
When I buy something I keep in mind the opinion of …	1.88 (0.77)	1.90 (0.73)	2.41	0.02
If I were worried about a friend starting to take drugs I would say a	3.02 (0.66)	2.83 (0.85)	3.39	0.00
When I am alone I look for the company of …	2.96 (0.80)	2.64 (0.97)	2.16	0.03
I can express my true feelings when I am with …	2.98 (0.74)	2.79 (0.90)	2.43	0.02
If I need to ask questions about sex I would turn to …	2.98 (0.77)	2.76 (0.92)	3.99	0.00
I understand and approve the reasons for …	2.49 (0.91)	2.10 (0.94)	2.31	0.02
I feel I can reveal my secrets …	3.20 (0.62)	3.03 (0.80)	2.07	0.04
I am often disappointed by …	2.90 (0.83)	2.70 (0.96)	−2.22	0.03

**Table 8:** The Independent Samples *t* Test: significant items of Teacher Relationship Scale.

Items	Middle School Students	High School Students	*t* Test for Independent Group	
	M (SD)	M (SD)	T	*p*-Value
I am truly understood by …	2.53 (0.75)	2.24 (0.75)	3.64	0.00
I enjoy spending time with …	3.03 (0.70)	2.60 (0.81)	5.23	0.00
If I were to dare bother the behavior of a friend I would say it to	2.65 (0.73)	2.42 (0.83)	2.75	0.01
I am treated with justice by …	3.06 (0.55)	2.91 (0.70)	2.29	0.02
When I buy something I keep in mind the opinion of …	2.18 (0.67)	1.99 (0.67)	2.58	0.01
When I am alone I look for the company of …	2.52 (0.75)	2.25 (0.79)	3.37	0.00
My happiness is greatly influenced by …	3.02 (0.52)	2.88 (0.74)	2.21	0.03
It is important for me to be paid by …	2.32 (0.75)	2.13 (0.74)	2.29	0.02
My values are similar to …	3.02 (0.57)	2.86 (0.77)	2.31	0.02
It is very close to my heart …	2.45 (0.79)	2.26 (0.74)	2.34	0.02

**Table 9:** MANOVA: influence of independent variables on relational ability.

Measures	Dependent Variables	F	*p*
Age	Peer Relationship	2.020	0.035
	Relationship with teachers	2.048	0.032
	Relationship with mother	2.229	0.019
	Relationship with father	2.047	0.032
Gender	Peer Relationship	8.001	0.005
	Relationship with teachers	0.027	0.870
	Relationship with mother	3.777	0.054
	Relationship with father	0.024	0.878
School Grade	Peer Relationship	2.632	0.107
	Relationship with teachers	2.314	0.130
	Relationship with mother	5.563	0.020
	Relationship with father	3.179	0.077
Age × Gender	Peer Relationship	1.526	0.144
	Relationship with teachers	1.796	0.073
	Relationship with mother	1.929	0.052
	Relationship with father	1.830	0.067
Age × School Grade	Peer Relationship	6.433	0.000
	Relationship with teachers	4.605	0.004
	Relationship with mother	5.722	0.001
	Relationship with father	5.003	0.002
Gender × School Grade	Peer Relationship	0.883	0.349
	Relationship with teachers	0.279	0.598
	Relationship with mother	0.004	0.951
	Relationship with father	0.029	0.865
Age × Gender × School Grade	Peer Relationship	4.617	0.004
	Relationship with teachers	8.795	0.000
	Relationship with mother	5.180	0.002
	Relationship with father	4.091	0.008

**Table 10:** Model summary of hierarchical regression analysis that predicts the school success.

Variables	R	Adjusted R	SE	Β	T	*p*
Emotional Ability	0.38	0.38	0.030	−0.472	−11.244	0.000
Environmental Control			0.022	0.160	3.857	0.000
Relational Skills			0.043	0.196	4.653	0.000

Abbreviation: β, beta standardized coefficients; SE, standard error.

**Table 11:** Model summary of hierarchical regression analysis that predicts school success.

Variables	R	Adjusted R	SE	Β	T	*p*
Emotional Ability	0.40	0.39	0.030	−0.445	−10.297	0.000
Environmental Control			0.022	0.151	3.622	0.000
Peer Relationship			0.042	0.105	2.401	0.017
Relationship with teachers			0.035	−0.060	−1.346	0.179
Relationship with mother			0.039	0.055	0.899	0.369
Relationship with father			0.038	0.175	2.890	0.004

Abbreviation: β, beta standardized coefficients; SE, standard error.

**Table 12:** Mediation effects summary.

Total Effect VI ≥ VD	Direct Effect VI ≥ VD	Relationship	Indirect Effect	Confidence Interval (CI)		t-Statistics	Conclusion
				Lower Bound Upper Bound			
0.3723 (0.000)	0.2179 (0.000)	H2 VI ≥ M1 ≥ M2 ≥ VD	0.1545; SE = 0.0341	0.0923	0.2258	4.530	Partial mediation

## Data Availability

Data supporting the conclusions of this article will be made available by the authors, without undue reservation.
